# Neuropsychological Testing and Machine Learning Distinguish Alzheimer’s Disease from Other Causes for Cognitive Impairment

**DOI:** 10.3389/fnagi.2017.00114

**Published:** 2017-04-25

**Authors:** Pavel Gurevich, Hannes Stuke, Andreas Kastrup, Heiner Stuke, Helmut Hildebrandt

**Affiliations:** ^1^Department of Mathematics, Free University of BerlinBerlin, Germany; ^2^Faculty of Science, Peoples’ Friendship University of RussiaMoscow, Russia; ^3^Department of Neurology, Municipal Hospital of Bremen-OstBremen, Germany; ^4^Department of Psychiatry and Psychotherapy, Charité–Universitätsmedizin BerlinBerlin, Germany; ^5^Department of Psychology, University of OldenburgOldenburg, Germany

**Keywords:** Alzheimer’s disease, MCI, total tau to Aβ(1–42) ratio, neuropsychological testing, dementia, machine learning

## Abstract

With promising results in recent treatment trials for Alzheimer’s disease (AD), it becomes increasingly important to distinguish AD at early stages from other causes for cognitive impairment. However, existing diagnostic methods are either invasive (lumbar punctures, PET) or inaccurate Magnetic Resonance Imaging (MRI). This study investigates the potential of neuropsychological testing (NPT) to specifically identify those patients with possible AD among a sample of 158 patients with Mild Cognitive Impairment (MCI) or dementia for various causes. Patients were divided into an early stage and a late stage group according to their Mini Mental State Examination (MMSE) score and labeled as AD or non-AD patients based on a post-mortem validated threshold of the ratio between total tau and beta amyloid in the cerebrospinal fluid (CSF; Total tau/Aβ(1–42) ratio, TB ratio). All patients completed the established Consortium to Establish a Registry for Alzheimer’s Disease—Neuropsychological Assessment Battery (CERAD-NAB) test battery and two additional newly-developed neuropsychological tests (recollection and verbal comprehension) that aimed at carving out specific Alzheimer-typical deficits. Based on these test results, an underlying AD (pathologically increased TB ratio) was predicted with a machine learning algorithm. To this end, the algorithm was trained in each case on all patients except the one to predict (leave-one-out validation). In the total group, 82% of the patients could be correctly identified as AD or non-AD. In the early group with small general cognitive impairment, classification accuracy was increased to 89%. NPT thus seems to be capable of discriminating between AD patients and patients with cognitive impairment due to other neurodegenerative or vascular causes with a high accuracy, and may be used for screening in clinical routine and drug studies, especially in the early course of this disease.

## Introduction

While the global number of patients with Alzheimer’s disease (AD) is increasing, recent clinical trials suggest for the first time the possibility of devising a disease-modifying treatment (among others, Sevigny et al., 2016). The early detection and differential diagnosis of AD is herefore becoming an urgent task. One early biomarker of AD is the ratio of the concentrations of total tau protein and beta amyloid (1–42), Aβ(1–42) in the cerebrospinal fluid (CSF; Frankfort et al., 2008; Hertze et al., 2010; van Rossum et al., 2010). A recent study demonstrated that these two proteins are the most important and earliest biomarkers for AD, even compared to PET (Palmqvist et al., 2016). However, since a lumbar puncture is an invasive and sometimes contraindicated method, it is crucial to develop valid and risk-free alternative methods for the diagnosis of AD.

Specific neuropsychological testing (NPT) might represent such a method, with a substantial body of evidence demonstrating its potential for differential diagnosis (Edmonds et al., 2015; Haanpää et al., 2015; Mansoor et al., 2015) and prognosis estimation (Landau et al., 2010; Peters et al., 2014). For identifying AD patients diagnosed by clinical criteria, it yielded an accuracy similar to CSF biomarkers (Schmand et al., 2010) and could predict the development of AD as early as 10 years before the clinical syndrome has developed (Boraxbekk et al., 2015; Mistridis et al., 2015). Moreover, since specific impairments in AD patients such as an increased number of false positives during recognition of semantically related or prototypical visual items (Gallo et al., 2004; Gold et al., 2007; Hildebrandt et al., 2009) are hitherto not covered by standard testing, the potential of NPT might not yet be fully deployed. While it thus seems that NPT might contribute to a risk-free early diagnosis of AD, it should be validated with established biomarkers like Aβ(1–42) and tau protein to reduce the risk of circular reasoning in predicting clinical diagnoses based on clinical neuropsychological assessments (Haldenwanger et al., 2010; Fields et al., 2011; Nelson et al., 2012, for previous work on relationships between NPT results and non-clinical biomarkers).

Furthermore, modern machine learning techniques have up to now only very rarely been used for the differential diagnosis of dementia based on the results of specific neuropsychological tests. However Klöppel et al. (2008) reported an effective differentiation between Alzheimer’s and frontotemporal dementia based on Magnetic Resonance Imaging (MRI) scans and machine learning and Wang et al. (2016) compared NPT and MRI scans as predictors for a machine-learning based distinction between Alzheimer’s and behavioral frontotemporal dementia. In general, these algorithms learn which specific neuropsychological deficit patterns are typical of AD patients in a training sample and then use these learned regularities to predict the AD status of new independent patients. During training, the algorithm is “blind” for the patient to be predicted and instead learns on all patients except the one to predict (leave-one-out technique). Thus, contrary to conventional analysis methods where the predicted cases are part of the training set, this approach resembles the clinical situation, in which the underlying pathology of a new patient has to be inferred based on his/her NPT results, arguably rendering the resultant predictions more meaningful (Lo et al., 2015). In the present study, we used the so-called “support vector classifier” (SVC), an established and powerful machine learning algorithm (Cristianini and Shawe-Taylor, 2000), for this purpose. Simplified, a SVC finds a separating hyperplane in the higher-dimensional data space (in our case, the NPT results) such that the classes (diagnoses) are separated with a maximal distance.

As outlined above, the main question of the present study was whether using more specific neuropsychological test batteries combined with machine learning might help to detect AD patients as defined by biomarkers, not by clinical criteria, and in a predictive manner (i.e., while separating training and test cases). To test the feasibility of this approach for a wide range of cognitive impairment severities, we included patients with minimal cognitive impairment (Mini Mental State Examination (MMSE) score lower than 29 but higher than 24) up until manifest dementia (MMSE score 24 and lower) and defined the presence of AD by a pathological Total tau/Aβ(1–42) ratio (TB ratio) in the CSF, which is still the most sensitive and specific biomarker for AD (Tapiola et al., 2009; Hertze et al., 2010; Palmqvist et al., 2016). This approach is in line with recently published research criteria, stating that the combination of cognitive impairment plus positive biomarker pattern confirms the diagnosis of a possible AD (Dubois et al., 2014). Separately for early patients with small cognitive impairment, late patients with marked cognitive impairment and the total sample comprising both groups, we assessed the predictive performance of machine learning based on standard and extended (“full”) NPT.

## Patients and Methods

### Patients

We recruited 158 inpatients with cognitive impairment from the neurological department of the hospital of Bremen-Ost. Patients with acute neurological causes for impairment (i.e., stroke or inflammatory diseases) were not included. To test if the diagnostic power of our approach might depend on the severity of cognitive impairment, we divided patients into an early group with MMSE scores between 25–28 and a late group with MMSE scores below 25 and report results separately for the two subgroups (alongside with the total group comprising all patients).

Demographic details and CSF biomarker values of the patients are summarized in Table 1. Patients were defined as AD patients according to the TB ratio in the CSF. For this purpose, we used the TB_pm_ ratio of 0.721 published by Tapiola et al. (2009; in line with this study, patients with a ratio above this TB_pm_ ratio were labeled as AD patients). According to Tapiola et al. (2009) the accuracy of the TB_pm_ ratio in predicting post-mortem analyzed neuropathology of AD is 85.4% with a specificity of 89.3% and a sensitivity of 84.2%. Based on these diagnostic labels, Table 1 also indicates which of the demographic and biomarker characteristics differed significantly between AD and non-AD patients.

**Table 1:** **Demographic information and results of cerebrospinal fluid analysis for the patients’ samples**.

	Early group (MMSE 25–28)		Late group (MMSE 8–24)	
Number of patients	89		69	
Sex*	Female: 30, Male: 59		Female: 42, Male: 27	
Number of patients with AD according to their TB ratio	AD: 23, non-AD: 66		AD: 47, non-AD: 22	
	Mean	*SD*	Mean	*SD*
Age (years)	69.8	7.4	71.4	6.8
Education	12.9	2.2	12.4	2.3
Total tau*	384.2	224.4	642.1	549.0
Aβ(1–42)*	788.5	317.7	518.9	233.5
Tau/Aβ(1–42) ratio*	0.61	0.55	1.65	1.71
Mini Mental Status Examination score*	26.4	1.1	19.7	4.3
Beck’s Depression Inventory score	9.8	8.1	10.6	8.4

*Significant (p < 0.05) difference between early and late group (chi-squared test for sex, t-test for all other variables). Abbreviations: MMSE, Mini Mental Status Examination; TB_pm_, Total tau/Aβ(1–42) ratio verified by Tapiola et al. (2009).

Of the 158 patients, 89 patients had a MMSE score between 25–28 (early group), and 69 patients between 8–24 (late group). Patients in the early group showed significantly higher Aβ(1–42) concentrations and lower total tau concentrations than patients in the late group (Table 1), but they did not differ in age, education and depressive mood. There was a significant difference in sex with female patients prevailing in the late group and male patients in the early group. Because of this difference and due to well-known gender-dependent effects in verbal and non-verbal cognitive abilities, we always performed classification separately for male and female patients (see below).

The study was approved by the Ethics committee of the University of Oldenburg and patients gave their informed consent to participate in the study.

### Neuropsychological Investigation

All patients underwent a neuropsychological examination, including the Consortium to Establish a Registry for Alzheimer’s Disease—Neuropsychological Assessment Battery (CERAD-NAB) test battery, which is standard for dementia testing and comprises a short version of the Boston Naming Test, semantic word fluency test, word list learning, figure copying and delayed figure recall (Morris et al., 1988, 1989). Moreover, the digit span from the Wechsler Memory Scale (Wechsler, 1987) was used to investigate verbal working memory performance and the Beck Depression Inventory (Beck and Bailer, 1985) to measure depressive symptoms.

Additionally, we carried out two newly developed neuropsychological tests aiming to capture cognitive impairments specific for AD patients. First, previous studies (Gold et al., 2007; Hildebrandt et al., 2009; Haldenwanger et al., 2010) have consistently demonstrated that specifically AD patients are suggestible for visually prototypical items and tend to rate them as already presented. To carve out this deficit, patients were first asked to name 16 pictures without knowing that they would later be asked to recognize them. After 15 min, 24 recognition trials were carried out. Each of these trials encompassed three pictures that stemmed from the same category to increase discrimination difficulty. Twelve of these triplets comprised pictures with biological content (eight triplets of animals, three triplets of body organs and one triplet with fruits), the other 12 triplets showed man-made items like vehicles, furnishings and instruments. As far as possible, the pictures were similar in their geometrical shape. We also ensured that the familiarity (frequency of use) of the target item was not higher than that of the two distractor items. Whereas sixteen of these recognition trials in fact comprised one of the initially shown pictures, eight of them only consisted of new pictures, increasing the probability of falsely positive responses. In each trial, the participants had to decide whether one of the three pictures had been shown before, and if so, which one. Based on our prior work (Hildebrandt et al., 2009), we expected that an increased number of false positives in this task would help to distinguish AD from other causes for cognitive impairment.

Second, AD patients show impairment in memory function but not in verbal understanding, which is typically impaired in frontotemporal and subcortical dementia (Rohrer et al., 2008). We therefore tested comprehension by giving the patients six written sets of instructions for constructional tasks (i.e., “draw a square, inside the square a circle and inside the circle a cross”) and counting the number of correct drawings (with a range from 0 to 6 correct solutions). The order of the six tasks was always the same and determined by their difficulty. Here, we expected that a comparatively intact functioning in this task should delineate AD patients from other causes.

### Neurological Investigation

The neurological investigation served to exclude acute causes for cognitive impairment and included medical history, physical and neurological examination, laboratory blood tests, brain imaging, electroencephalography and a lumbar puncture.

The blood sample analysis comprised blood count, erythrocyte sedimentation rate, electrolytes, creatinine, urea, transaminases, blood glucose, TSH, C-reactive protein, vitamin B 12 and folic acid.

### Determination of tau Protein and Aß(1–42)

Lumbar punctures were performed during the same hospital stay as the NPT and carried out by a trained neurologist, using a 22-gauge Sprotte spinal needle. Approximately 5 ml CSF were taken. CSF samples were collected in polypropylene tubes and transported to an adjacent laboratory within 30 min. CSF samples were analyzed for cell count, total protein, lactate, glucose, IgG, IgA, IgM, borreliose antibodies, Aβ(1–42) and total tau protein. Total tau was determined using a commercial sandwich enzyme-linked immunosorbent assay and Aβ(1–42) using a sandwich ELISA (Innotest^®^ hTAU-Ag and Innotest^®^ β-amyloid (1–42), respectively, Innogenetics, Belgium). All tests were performed at the Medizinisches Labor Bremen.

### Classification Procedure

The main goal of our study was to predict if cognitive impairment in a single patient is due to AD or other causes based on specific patterns of neuropsychological test results that have to be inferred by the algorithm from the remaining patients without the patient to be predicted. To this end, we used the SVC (Cristianini and Shawe-Taylor, 2000), in a leave-one-out procedure comprising the following steps:

1. Assign objective diagnostic labels to *N* patients: a patient belongs to class 1 (AD patients) if the TB ratio is greater than the post-mortem validated TB_pm_ ratio.
2. Remove the to-be-predicted patient from the data (leave-one-out): for the *i*-th patient from the target subgroup (*i* = 1,…, *N*): remove from the data matrix *X* the *i*-th row corresponding to the *i*-th patient.
3. Preprocessing of the data (normalization and feature reduction): normalize the resulting matrix *X*_i_ by centering each column to its mean and scaling to unit variance; apply principal component analysis (PCA, feature reduction) to *X*_i_.
4. Train the algorithm on the data set without the to-be-predicted patient: fit the SVC, using *X*_i_. In order to avoid artificially high accuracy values in the case of an unbalanced number of cases per class (i.e., to avoid a high sensitivity at the expense of poor specificity or vice versa (Nguyen et al., 2009)), the algorithm was trained to obtain a maximal harmonic mean between sensitivity and specificity. The harmonic mean between sensitivity and specificity is defined as 2*sensitivity*specificity/(sensitivity + specificity).
5. Predict the patient: apply the transformations from step 3 to the row corresponding to the *i*-th patient; predict with the fitted SVC to which class the *i*-th patient belongs.
6. Repeating step 2–5 for all *i* = 1,…, *N* and comparing the prediction with the pre-classification from step 1, find the confusion matrix for the target subgroup of *N* patients.

The leave-one-out algorithm was employed with the explained variance of the PCA *E* = 0.7, 0.8, 0.9, 0.99, 1. We used the *E* that yielded the maximal harmonic mean of sensitivity and specificity. As explained above (see paragraph “Patients”), classification was carried out separately for male and female patients and the results reported below refer to the pooled outcomes of both sexes.

### Classification Superiority over Conventional Testing

Additionally to obtaining absolute classification accuracies, we aimed at demonstrating that AD could superiorly be delineated from other causes for cognitive impairment when the new specific neuropsychological tests (suggestibility for visually prototypical items and verbal understanding as described above) were included in the test battery. To this end, we used the binomial test to compare the number of correctly classified patients according to a standard neuropsychological model that comprised gender, education, age and all CERAD-NAB scores as predictors (standard model) with that according to the full test battery that additionally included new tests (full model). Note that a patient was considered as correctly classified if the prediction of the algorithm matched the “objective” label based on his/her TB ratio. This analysis thus tests if the classification results obtained when using the full test battery including the new tests as predictors were significantly superior to the results using demographic variables plus standard NPT only.

## Results

### Neuropsychological Tests Results

Table 2 shows the neuropsychological test results for the three groups. Note that all patients were cognitively impaired, and hence AD and non-AD groups are exclusively defined by the TB_pm_ ratio. Cognitive performance differed between AD and non-AD patients depending on disease stage: in the early group, patients with AD-like TB_pm_ ratio scored significantly better in language comprehension and working memory, whereas in the late and total group, tests on memory performance showed the typical impairments for the AD patients. These results indicate that the most sensitive combination of neuropsychological tests for distinguishing AD from non-AD patients depends on the disease stage.

**Table 2:** **Neuropsychological test results of the patients’ groups**.

	Early group (MMSE 25–28)				Late group (MMSE 8–24)				Total group			
	Non-AD		AD		Non-AD		AD		Non-AD		AD	
	Mean	*SD*	Mean	*SD*	Mean	*SD*	Mean	*SD*	Mean	*SD*	Mean	*SD*
*Newly developed tests*
Visual recognition, omissions	2.6	2.1	2.6	2.3	3.9	3.0	3.9	3.9	3.0	2.4	3.5	3.5
Visual recognition, false positives^*,~^	1.1	1.9	2.0	2.2	2.8	3.1	5.1	4.3	1.5	2.3	4.1	4.0
Language comprehension^+,~^	2.7	1.5	4.3	1.2	3.0	1.7	3.5	1.7	2.8	1.6	3.7	1.6
*CERAD-NAB*
Semantic wordfluency	13.0	4.4	15.0	4.4	10.8	5.2	9.6	4.9	12.4	4.7	11.3	5.3
Boston naming	13.0	2.3	13.6	1.6	12.3	2.0	12.5	4.1	12.8	2.2	12.8	3.6
Wordlist learning^*,~^	13.0	3.4	13.3	4.0	10.4	2.8	8.2	4.3	12.3	3.5	9.8	4.8
Wordlist delayed recall ^*,~^	3.8	1.9	3.3	1.6	2.4	1.7	1.4	1.5	3.5	1.9	2.0	1.8
Wordlist savings^~^	69.3	30.9	64.5	27.4	48.5	33.5	36.2	40.0	64.1	32.7	45.3	38.6
Wordlist discrimination^*,~^	92.0	8.7	89.1	10.4	85.0	9.5	78.2	14.1	90.2	9.4	81.6	13.9
Visuoconstruction	8.9	1.8	9.4	1.8	8.8	1.8	8.6	1.9	8.9	1.8	8.8	1.9
Visuoconstruction delayed recall^~^	5.7	3.2	4.7	2.7	3.7	2.5	2.5	2.8	5.2	3.1	3.2	2.9
Visuoconstruction savings ^*,~^	63.4	33.9	50.8	28.2	42.1	30.0	25.8	28.7	58.1	34.1	33.7	30.7
*Working memory testing*
Digit span forward^+^	31.8	24.2	50.2	29.7	35.0	29.4	30.8	28.5	32.6	25.5	36.9	30.1
Digit span backward^+^	19.7	17.9	37.9	24.7	19.3	26.2	15.3	15.4	19.6	20.1	22.5	21.5

^+^Early group: p < 0.05 Alzheimer’s disease (AD) vs. non-AD, two-sample t-Test. *Late group: *p* < 0.05 AD vs. non-AD, two-sample t-Test. ^~^Total group: p < 0.05 AD vs. non-AD, two-sample t-Test. Abbreviations: MMSE, Mini Mental Status Examination. With the exception of visual recognition, omissions and visual recognition, false positives, higher values indicate a more intact functioning in the respective test.

### Classification Results

Table 3 summarizes the results of predicting the cause for cognitive impairment of the patients (AD or non-AD defined by the TB_pm_ ratio), using the full battery of neuropsychological tests (column “full”) and using only the standard testing (column “standard”). Classification accuracy was 82% in the total group with better results for the early group (89%) compared to the late group (72%). This decline in accuracy with progressing disease was mainly due to decreasing specificity, whereas sensitivity figures remained largely unchanged. Hence, in the late stage, non-AD causes for cognitive decline increasingly resemble the pattern of cognitive deficits that allowed precise delineation of AD at earlier stages and are therefore misclassified as AD.

**Table 3:** **Sensitivity, specificity, their harmonic mean and accuracy of predicting AD (according to TB ratio) for the three MMSE groups and the two models (full model with and standard model without newly developed tests)**.

	Early group: MMSE 25–28 (89 patients)		Late group: MMSE 8–24 (69 patients)		Total group: MMSE 8–28 (158 patients)	
Model	Full	Standard	Full	Standard	Full	Standard
Sensitivity	0.74	0.48	0.79	0.70	0.77	0.63
Specificity	0.94	0.82	0.59	0.50	0.85	0.73
Harmonic mean	0.83	0.60	0.68	0.58	0.81	0.67
Accuracy	0.89	0.73	0.72	0.64	0.82	0.68
Confusion	[17 6]	[11 12]	[37 10]	[33 14]	[54 16]	[44 26]
matrix	[4 62]	[12 54]	[9 13]	[11 11]	[13 75]	[24 64]

*The confusion matrix denotes the number of patients correctly classified by Support Vector Classifier (SVC) to have AD (true positives, top/left), the number of patients incorrectly classified by SVC to have no AD (false negatives top/right), the number of patients incorrectly classified by SVC to have AD (false positives, bottom/left) and the number of patients correctly classified by SVC to have no AD (true negatives, bottom/right). Abbreviations: MMSE, Mini Mental Status Examination; TB_pm_, Total tau/Aβ(1–42) ratio verified by Tapiola et al. (2009)*.

### Classification Superiority over Conventional Testing

Table 3 also provides a comparison between prediction performance based on the neuropsychological standard testing compared to the full set with the newly developed tests. Formal comparison using binomial tests revealed a significant classification superiority of the full model with *p* < 0.001 for the total and the early group and a trend-wise superiority in the late group (*p* = 0.071). Hence, the accuracy achieved by the full model that added new specific neuropsychological tests was significantly better than that achieved by the standard testing in the early and total group, and also trend-wise in the late group.

## Discussion

With the advent of new treatments, early detection of AD becomes an urgent task. We investigated the potential of NPT to detect AD patients (defined by an AD-typical protein pattern in the CSF) among patients with cognitive impairment caused by various etiologies. We found that in the total group, 82% of the patients could be correctly classified as AD or non-AD by these means.

The accuracy of AD identification depended on disease stage with a better accuracy (89%) for early stage patients (MMSE 25–28) compared to late stage patients (MMSE <25, 72% accuracy). A conceivable explanation for this discrepancy might be that the disease progress entails a more *global* cognitive impairment, which may impede the detection of more specific and fine-grained cognitive performance differences. Consistent with this, a prior study using a similar test battery showed that whereas Mild Cognitive Impairment (MCI) patients with possible AD and with Parkinson’s disease differed in several tests, fewer tests were able to distinguish between *demented* AD and Parkinson’s disease patients (Hildebrandt et al., 2013).

Part of our neuropsychological assessment was the CERAD-NAB, a test battery frequently used in memory clinics. A recent prospective study showed that the CERAD-NAB is able to predict the development of MCI during the next 8 years and conversion to AD after 10 years (Mistridis et al., 2015). The predictive power of the CERAD-NAB for diagnosis based on clinical criteria is thus established and also a correlation of its items (wordlist learning) with CSF biomarkers could be shown (Haldenwanger et al., 2010). Nevertheless, in our study, classification of biomarker-based diagnoses was significantly less accurate using the CERAD-NAB alone than classification using CERAD-NAB plus the newly developed tests. Thus, the newly added assessments of language comprehension performance and of false positives in visual recognition improved the classification performance, speaking for the general possibility to increase the diagnostic power of NPT by using more specific task components.

One strength of this study is that we did not preselect for specific cognitively impaired patients, but included patients with various clinical diagnoses: this is a scenario which comes closest to clinical routine and is different from previous work that either differentiated AD dementia patients from healthy controls (e.g., Zhang et al., 2011) or from one particular differential diagnosis (Klöppel et al., 2008; Wang et al., 2016). Moreover, we classified our patients exclusively by the TB ratio obtained 1–2 weeks after NPT, implying an effective blinding of the diagnosis for the investigator. Another strength is the use of the SVC for prediction of the patients’ status. The SVC simulates the clinical routine situation—predicting the diagnosis of a given patient on the background of the scientific and clinical knowledge available up to the moment of his or her assessment. It avoids overfitting, because the predicted patient is not part of the training set for the model as in standard discriminant or regression analyses and it does not presuppose normally distributed data.

An accuracy between 75% and 89% is an encouraging result, considering that it refers to the *differential* diagnosis of AD in a sample of cognitively impaired individuals. For distinguishing between etiologically defined dementias, the accuracy based on CSF or MRI biomarkers alone is typically lower than these figures (Struyfs et al., 2015; Wang et al., 2016). For the specific purpose of identifying patients with preclinical AD, it is important to note that classification in our early disease sample showed superior results with an accuracy of almost 90%. Future work on potential clinical translations of these findings could take up four different lines of research: first, try to further improve the discriminative power of the test battery by developing neuropsychological tests with higher sensitivity for AD typical aspects of memory impairment. Second, try to streamline the test battery by removing tests that did not significantly contribute to the predictive power and thereby facilitate the clinical application. Third, validate the results in an independent patient collective. Fourth, explore the synergistic potential of combinations of MRI, lumbar puncture and NPT for a maximal accurate diagnosis of AD. From a methodological point of view, it would be worthwhile to also test non-linear classification techniques like neural networks to tackle the resulting high dimensional data sets for this purpose.

Obviously, there are also certain limitations: our study is a single center study and the number of patients is not very large, especially after sub-grouping. Moreover, gold standard of AD diagnosis is the neuropathological investigation of postmortem brain tissue that was not employed in our study. Thus, although being in line with diagnostic guidelines (Dubois et al., 2014), our external biomarker criterion for the diagnosis of AD was not perfect and showed 15% disagreement with post-mortem results in prior work (Tapiola et al., 2009). Nevertheless, our results suggest that NPT combined with properly trained machine learning algorithms might help to improve differential diagnosis of AD and to preselect patients for future drug studies with a safe and easy method.

## Author Contributions

HH and AK: design of the study; data collection. PG, HaS, HH and HeS: statistical analyses. HeS, HH and PG: writing of the article. PG, HaS, AK, HeS and HH: article correction and final approval.

## Funding

The research project received no external funding.

## Conflict of Interest Statement

The authors declare that the research was conducted in the absence of any commercial or financial relationships that could be construed as a potential conflict of interest.

## Abbreviations

Aβ(1–42): Beta-Amyloid(1–42)
AD: Alzheimer’s Disease
CERAD-NAB: Consortium to Establish a Registry for Alzheimer’s Disease—Neuropsychological Assessment Battery
MCI: Mild Cognitive Impairment
MMSE: Mini Mental Status Examination
MRI: Magnetic Resonance Imaging
NPT: Neuropsychological Testing
PCA: Principal Component Analysis
SVC: Support Vector Classifier
TB ratio: Total tau/Aβ(1–42) ratio
TB_pm_ ratio: Total tau/Aβ(1–42) ratio of 0.721 post-mortem verified by Tapiola et al. (2009).
